# Magnesium from Deep Seawater as a Potentially Effective Natural Product against Insulin Resistance: A Randomized Trial

**DOI:** 10.3390/medicina60081265

**Published:** 2024-08-05

**Authors:** Ji Yeon Ham, You Kyung Jang, Byong Yeob Jeon, Yun Hee Shon

**Affiliations:** 1 Department of Laboratory Medicine, Kyungpook National University Chilgok Hospital, School of Medicine, Kyungpook National University, 807 Hogukro Buk-gu, Daegu 41404, Republic of Korea; 2 QBM Research Institute, QBM Co., Ltd., 7-25 Gangnam-daero 27-gil, Seocho-gu, Seoul 06752, Republic of Korea; 3 Bio-Medical Research Institute, Kyungpook National University Hospital, 135 Dongdukro Jung-gu, Daegu 41940, Republic of Korea

**Keywords:** clinical trial, seawater, diabetes mellitus, insulin resistance, magnesium

## Abstract

*Background and Objectives*: Deep seawater has been shown to restore pancreatic function in obese diabetic mice and considerably improve the homeostatic model assessment for insulin resistance, total cholesterol, and low-density lipoprotein cholesterol concentrations in patients with impaired fasting glucose or glucose tolerance. In this study, the effect of 12-week daily consumption of magnesium (Mg^2+^)-containing deep seawater mineral extracts on blood glucose concentration and insulin metabolism-associated indicators was investigated in patients with impaired glucose tolerance. *Materials and methods*: In this 12-week randomized, double-blind trial, patients (*n* = 37) with impaired glucose tolerance consumed deep seawater mineral extracts. Changes in blood glucose concentration and related indicators were compared between the treatment group and placebo group (*n* = 38). *Results*: The fasting insulin, C-peptide, homeostatic model assessment for insulin resistance, quantitative insulin sensitivity check index, homeostatic model assessment of beta-cell function, and Stumvoll insulin sensitivity index values in the deep seawater mineral extract group showed improvements compared with the placebo group. However, no significant differences between groups were observed in fasting blood glucose, postprandial blood glucose, glycated hemoglobin, or incremental area under the curve values. *Conclusions*: Oral supplementation with deep seawater mineral extracts enriched in Mg^2+^ markedly improves insulin sensitivity in patients with pre-diabetes. This study illustrates the potential clinical application of natural Mg^2+^ from deep seawater to alleviate insulin resistance in patients with pre-diabetes. *Trial registration*: This trial was retrospectively registered with Clinical Research information Service (CRIS), No. KCT0008695, on 8 August 2023.

## 1. Introduction

Diabetes is characterized by dysglycemia and is a major contributor to the development of stroke, heart attacks, and blindness. Considering the rapidly increasing global incidence of diabetes, the International Diabetes Federation predicts an alarming global health situation owing to diabetes and its associated complications in the 21st century [1].

The pre-diabetic stage is characterized by insulin metabolism abnormalities, pancreatic dysfunction, and intermediate hyperglycemia [2]. Approximately 70% of people with pre-diabetes develop diabetes [3]. Given that pre-diabetes increases the risk of microvascular and macrovascular complications [4,5], individuals with pre-diabetes risk should be identified early, and lifestyle changes, such as diet control, regular physical exercise, and pharmacological intervention to prevent further progression and associated complications, should be implemented [6,7].

Insulin resistance (IR) is a major cause of type 2 diabetes (T2D) that increases the risk of subsequent retinopathy, renal dysfunction, and cardiovascular diseases. Within patients with pre-diabetes, the insulin level is elevated to help maintain normal glucose levels. However, this can lead to chronic hyperinsulinemia, hyperglycemia-induced pancreatic beta-cell dysfunction, and ultimately T2D. Obesity, increased visceral adipose tissue, family history of T2D, and fatty liver are associated with IR. Although the mechanisms underlying IR are not completely understood, ectopic lipid accumulation, endoplasmic reticulum stress, and inflammation of the liver and skeletal muscles are suggested as major contributors [8].

Various compounds have been tested for blood glucose regulation in patients with diabetes or individuals with pre-diabetes [9,10,11]. Oral magnesium (Mg^2+^) supplements can improve blood glucose control in individuals with pre-diabetes and patients with diabetes [12,13,14]. Mg^2+^ is vital in glucose metabolism, fat, protein, and nucleic acid synthesis, and muscle contraction and is a key factor influencing dyslipidemia, hypertension, metabolic abnormalities, and bone health [15]. Additionally, interactions between Mg^2+^ and calcium (Ca^2+^) affect IR at the cellular level [16]. Several clinical studies have reported that dietary supplementation of Mg^2+^ alleviates IR and reduces blood glucose levels [17,18,19]. Low Mg^2+^ levels are common in patients with T2D; this is associated with reduced metabolic rate and chronic complications [20,21]. Therefore, Mg^2+^ supplementation is suggested as an adjuvant therapy for managing diabetes [13,14].

Seawater at depths of 200–2300 m is referred to as deep seawater, where almost no organic matter or pathogens exist and a temperature of <3 °C is maintained. Deep seawater is rich in minerals vital for the growth of marine organisms, including sodium (Na^+^), Mg^2+^, Ca^2+^, and potassium (K^+^) [22], making it a useful marine resource for the food, medical, cosmetic, and agriculture industries.

Preliminary animal studies have shown that deep seawater can restore pancreatic function in obese diabetic mice fed a high-fat diet [23]. Additionally, deep seawater lowers blood glucose levels by downregulating the expression of glucose production-associated genes and upregulating the expression of glucose uptake- and fatty acid degradation-associated genes [23,24,25]. In a previous eight-week clinical trial involving patients with impaired fasting glucose or impaired glucose tolerance (IGT), homeostatic model assessment for IR (HOMA-IR), total cholesterol, and low-density lipoprotein cholesterol levels improved following administration of a deep seawater formulation containing Mg^2+^ as a functional ingredient [26]. These findings demonstrate the positive effects of Mg^2+^ content in deep seawater on blood glucose metabolism, including IR alleviation.

In this study, we aim to confirm the IR-alleviating effect of Mg^2+^-containing deep seawater by measuring changes in various indicators associated with insulin metabolism. Further, we evaluate the levels of glucose and blood glucose-related indicators in patients with IGT following the administration of Mg^2+^-containing deep seawater mineral extracts for 12 weeks.

## 2. Materials and Methods

### 2.1. Ethical Considerations

This trial was approved by the Institutional Review Board of Jeonbuk National University Hospital, Republic of Korea (IRB No. CUH 2021-06-075-001, date 4 September 2021). It was performed at the Functional Food Clinical Trial Center of Jeonbuk National University Hospital according to the tenets of the Declaration of Helsinki and in accordance with the Good Clinical Practice guidelines. Clinical trial progress was monitored at the hospital by Bio Food Story Co., Ltd. (16 Angol 4-gil, Deokjin-gu, Jeonju-si, Jeollabuk-do, Republic of Korea), a contract research organization. After sufficiently explaining the purpose and contents of the clinical trial and the effects and adverse effects of functional foods to the participants, their voluntary written consent was obtained (Informed Consent Document, ICD). This clinical trial was registered at cris.nih.go.kr under Clinical Trial Registration Number KCT0008695.

### 2.2. Participants

Participants who met all inclusion criteria and did not meet any of the exclusion criteria were selected for this trial (Table S1). The study included adult males and females aged 19–70 years at the time of screening with a 2 h blood sugar level of 140–199 mg/dL on a 75 g oral glucose tolerance test (OGTT). Only those who provided written informed consent and agreed to follow the guidelines (ICD) after receiving a detailed explanation and expressing understanding were enrolled. The exclusion criteria are listed in Table S1. 

The following assumptions were made to calculate the number of participants. A two-sided test was used as the statistical hypothesis test to evaluate the variable. At a significance level of 5%, the type II error was set to 0.2 to maintain 80% test power. The ratio of the number of test examples in the test group and the placebo group was 1:1. Based on the results of Rodriguez-Morán and Guerrero-Romero [20], the difference in the average change in blood glucose level at 2 h in the 75 g OGTT between the intake groups was assumed to be 17.80 mg/dL, and the standard deviation was assumed to be 25.47 mg/dL. Under such conditions, the number of participants required for trials was calculated using the method described by Sakpal [27], with a minimum of 32 participants per group. Considering a dropout rate of 20%, the number of participants required for an effective evaluation was approximately 40 per group; a total of 80 participants were enrolled in the trial. 

The patient group included in the main analysis comprised individuals who ingested the product for the clinical trial at least once with a full analysis set (FAS), underwent efficacy evaluation at least once, and did not violate the key inclusion criteria. The decision regarding the analysis group was made through a blind review meeting. In the FAS, 75 patients (93.7%) were included, excluding one test group patient who took contraindicated drugs and four patients (two in the control group and two in the test group) whose data were unavailable after randomization of the participants. The final control group comprised 38 patients (96.0%), and the test group comprised 37 patients (92.5%). Efficacy was evaluated by comparing the changes in intake at 6 and 12 weeks with baseline measurements between and within the intake groups.

### 2.3. Clinical Trial Product

Deep seawater was collected from a depth of 1500 m at a point approximately 7 km away from Hyeonpo-ri, Buk-myeon, Ulleungdo (Republic of Korea) and passed through a microfiltration membrane (Toray Advanced Materials Korea Inc., Seoul, Republic of Korea). The filtered raw water was concentrated through reverse osmosis (LG Chem Ltd., Seoul, Republic of Korea) and vacuum-evaporated (RDF Ltd., Seoul, Republic of Korea) to produce concentrated deep seawater with Mg^2+^ as the main component. The concentrated water was spray-dried, and the powdered (Eins System Co., Ltd., Seoul, Republic of Korea) deep seawater mineral extracts were used as the primary raw material for the clinical trial products. Inductively coupled plasma-optical emission spectrometry (ICP-OES, Thermo Fisher Scientific Inc., Waltham, MA, USA) was used for quantitative mineral analysis of deep seawater mineral extracts (Table 1).

The product used in the clinical trial was in an aqueous state, and the ingredients and contents per bottle (60 mL) are listed in Table 1. The control product (placebo) was prepared using purified water as the main component, and its weight and calories were almost identical to those of the clinical trial product (Table 1).

The functional component of the trial product was Mg^2+^, and the ingested amount was set based on a previous clinical trial [26]. Specifically, 350 mg of Mg^2+^ was consumed by each individual, corresponding to the recommended daily intake of 350 mg of Mg^2+^ in the Korean dietary standard. It was also within the range for currently marketed Mg^2+^ supplement products (300–500 mg per day). The clinical trial product was consumed twice daily (one bottle, 60 mL) for 12 weeks.

### 2.4. Study Design

This 12-week, randomized, double-blind trial evaluated the effects of oral ingestion of deep seawater mineral extracts containing Mg^2+^ on blood glucose concentration and related indicators. Patients with IGT consumed deep seawater mineral extracts for 12 weeks; the changes in blood glucose and related indicators were investigated and compared with those of patients who consumed a placebo. A CONSORT flowchart (Figure S1) and a checklist (Table S2) are provided.

**Figure 1:**
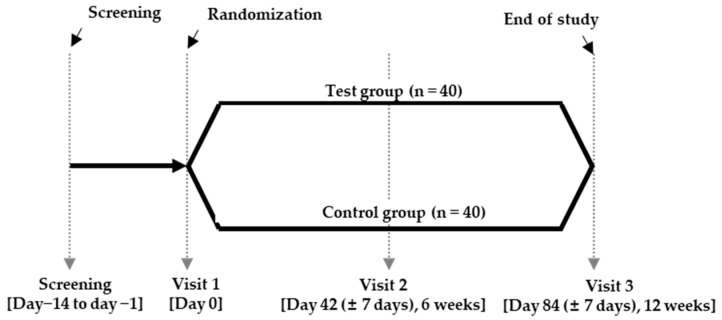
Clinical trial design. Evaluation of the effects of deep seawater mineral extract on blood glucose levels and glucose-related parameters.

**Figure 2:**
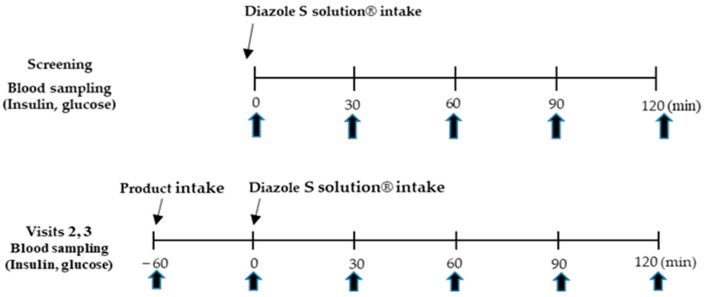
Seventy-five-gram oral glucose tolerance test performed during screening and at the second and third visits. OGTT, oral glucose tolerance test.

This randomized trial was performed as previously reported [26]. The clinical trial design is illustrated in Figure 1, and a schedule summary is presented in Table S3. Blood glucose-related indicators were measured, and the effectiveness of the deep seawater mineral extracts containing Mg^2+^ was assessed. For blood glucose-related tests, 75 g OGTT, incremental area under the curve (iAUC)_0–2h_, C-peptide, glycated hemoglobin, type A1c (HbA1c), and surrogate markers of IR were measured. The study participants fasted for at least 12 h; the 75 g OGTT was performed during screening and at the second and third visits. The test method performed during screening involved ingesting 75 g of glucose solution (Diazole S solution^®^) (Taejoon Pharmaceutical Co. Ltd., Seoul, Republic of Korea) [28] within 5 min (0 min) of drawing blood (0 min) for fasting blood glucose and fasting insulin tests. Blood was collected 30, 60, 90, and 120 min after taking 75 g of glucose solution (Figure 2). The 75 g OGTT conducted at the second and third visits involved blood collection before product intake, product intake (−1 h), 1 h after product intake (0 min), and intake of the 75-g glucose solution (Diazole S solution^®^). Blood collection was conducted at 30, 60, 90, and 120 min after glucose solution ingestion (Figure 2). All blood samples used for the examination were discarded after testing.

iAUC_0–2h_ is the only area higher than the baseline level based on the blood sugar concentration among all time points (0, 30, 60, 90, and 120 min) at which 75 g OGTT was performed. In principle, the levels of C-peptide and HbA1c should be measured using blood drawn after fasting for at least 12 h. The collected blood was discarded after analysis. HOMA-IR [29], homeostatic model assessment of β-cell function (HOMA-β) [29], quantitative insulin sensitivity check index (QUICKI) [30], insulin sensitivity index (ISI)_stumvoll_ [31], and ISI_0,120_ [32] were evaluated as insulin surrogate indicators.

Serum magnesium content was measured using an Atellica CH Mg^2+^ assay on a Siemens Atellica CH Analyzer (Siemens Healthcare Diagnostics Inc., Tarrytown, NY, USA) [33].

### 2.5. Statistical Analysis

SAS 9.4 (SAS Institute, Cary, NC, USA) for Windows was used for statistical analyses. The data obtained from the clinical trial are presented as descriptive statistics, such as mean and standard deviation (SD), and the significance of distinction was set at *p* < 0.05 with a two-sided test. For the homogeneity test between the groups and the baseline homogeneity test, the chi-square test, Fisher’s exact test, or independent *t*-test were used. Analysis of covariance or sub-analysis was performed by correcting demographic information items that were not homogeneous with items considered to affect the efficacy evaluation (e.g., hypertensive medications and anthropometric indicators) as covariates. When analyzing the efficacy of evaluation variables of the participants included in the FAS, missing values after baseline were replaced with the last observation carried forward for analysis.

Regarding the patient demographic information, descriptive statistics for each intake group were presented for all randomly assigned participants (intention-to-treat), and a statistical test was performed to detect differences in means or proportions. The degree of change after 6 and 12 weeks of intake compared with the baseline values within the intake group was analyzed using a paired *t*-test or the Wilcoxon signed-rank test based on normality fulfillment. Changes between the intake groups after 6 and 12 weeks compared to the baseline values were analyzed by applying an independent *t*-test or the Wilcoxon rank-sum test based on normality fulfillment.

## 3. Results

### 3.1. Participants

The target number of participants for this clinical trial was 80, and the number of participants for termination was 64. A total of 185 volunteers underwent a screening test after providing written informed consent, and 80 participants were deemed suitable based on the inclusion criteria. The participants enrolled in the clinical trial were randomly and double-blindly allocated to two groups (*n* = 40 each for control and test groups). Nine participants (control: 3, test: 6) dropped out during the clinical trial. Therefore, 71 participants completed all procedures. The actual status of participation in the study, current status, and reasons for dropping out are summarized in Figure S1.

The demographic characteristics of all participants (i.e., 80 people) were analyzed to confirm the validity of the random assignment (Table 2). No significant difference was observed between the intake groups; therefore, the randomization of participants in the present study was assessed to be relatively appropriate. 

Regarding the intake status of the product for the clinical trial, the degree of compliance was assessed after collecting the remaining amount of product returned by the participants at each visit. The product intake status and compliance of the participants are shown in Table 3. None of the patients were excluded from the analysis because the overall compliance exceeded 70%. The control and test groups showed 95.09 ± 5.65% and 94.40 ± 5.25% compliance, respectively, with no significant differences between the groups (*p* = 0.5993).

Dietary intake was analyzed using the dietary record written by the participant before intake of the product (baseline), after 6 weeks of intake (second visit), and after 12 weeks of intake (third visit). There was no significant change within or between intake groups after 6 and 12 weeks compared to the baseline (*p* > 0.05). This observation ruled out the effect of dietary amounts on the results (Table 4).

Alterations in the degree of physical activity during the clinical trial were also analyzed. On the day of screening (as well as the second and third visits), the physical activity level (metabolic equivalent task (MET)) was determined using the Global Physical Activity Questionnaire completed by the participants. No significant difference was found between the placebo and test groups at weeks 6 and 12 compared to baseline (*p* > 0.05; Table 5), suggesting that physical activity status did not influence the metabolic changes observed.

### 3.2. Efficacy Evaluation

No significant difference was observed between the intake groups in the 75 g OGTT conducted before intake (baseline) (Table 6, *p* > 0.05). The blood glucose concentration at 2 h (PPG_2h_) and iAUC_0–2h_ showed no significant differences between the test and placebo groups from baseline to 6 and 12 weeks after intake (*p* > 0.05), respectively.

No significant differences were observed between the groups (*p* > 0.05) in the fasting blood glucose or postprandial blood glucose concentrations after 6 and 12 weeks of intake in the 75 g OGTT compared with the baseline concentration (30-min (PPG_0.5h_), 60-min (PPG_1h_), and 90-min (PPG_1.5h_)) (Table 6). 

No significant baseline differences were observed between the intake groups, C-peptide, or insulin concentrations in the 75 g OGTT conducted before intake (baseline) (*p* > 0.05). After 12 weeks of intake, the fasting insulin concentrations decreased by 0.22 ± 3.93 μU/mL in the control group and 2.47 ± 4.51 μU/mL in the test group, showing a significant difference in variation between the intake groups compared with the baseline concentration (*p* < 0.05). The C-peptide concentration also decreased by 0.12 ± 0.51 ng/mL after 12 weeks of intake compared with the baseline concentration in the test group, whereas it increased by 0.21 ± 0.70 ng/mL in the placebo group, showing a significant difference between the intake groups (*p* < 0.05). No significant difference was found in the variation of insulin concentrations at 30, 60, or 90 min from the baseline concentrations after 6 or 12 weeks of intake in the 75 g OGTT (*p* > 0.05; Table 7).

The IR index (HOMA-IR), insulin secretion capacity index (HOMA-β), insulin sensitivity indices (QUICKI, ISI_0,120_, and ISI_stumvoll_), and HbA1c concentration were evaluated within and between intake groups (Table 8).

The HOMA-IR index increased by 0.42 ± 1.72 and 0.59 ± 1.48 in the placebo group after 6 and 12 weeks of intake, respectively, compared with the baseline values. In contrast, it decreased by 0.37 ± 1.40 and 0.29 ± 1.40, respectively, in the test group; a significant difference in HOMA-IR was detected between the intake groups after 6 and 12 weeks of intake (*p* = 0.0316 and *p* = 0.0102, respectively).

The value of HOMA-β—an indicator of insulin secretion ability—increased by 13.24% ± 50.52% after 12 weeks of intake compared with the baseline in the placebo group, whereas it decreased by 15.61% ± 44.62% in the test group, demonstrating a significant difference between intake groups (*p* = 0.0107).

The value of QUICKI—an insulin sensitivity index—decreased by 0.01 ± 0.03 in the placebo group and increased by 0.00 ± 0.03 in the test group after 12 weeks of intake compared with the baseline value, demonstrating a significant difference in the variation between the intake groups (*p* = 0.0378). The ISI_stumvoll_ also significantly differed between the intake groups after 6 and 12 weeks of intake compared with the baseline value (*p* = 0.0073); however, no significant differences were observed in ISI_0, 120_ between the intake groups (*p* > 0.05).

The HbA1c levels measured before intake (baseline) demonstrated no difference in baseline levels between the intake groups (*p* > 0.05). The HbA1c levels significantly decreased after 12 weeks of intake compared with the baseline level in the test group (*p* < 0.05); however, no significant difference was observed between the intake groups (*p* > 0.05).

## 4. Discussion

This study explored the efficacy of deep seawater mineral extracts on indicators associated with blood glucose level and insulin sensitivity following daily consumption for three months by patients with IGT. After three months of intake, the fasting insulin, C-peptide, HOMA-IR, HOMA-β, QUICKI, and ISI_stumvoll_ levels in the deep seawater mineral extract group were considerably improved compared with those in the placebo group. However, no significant difference was observed in fasting blood glucose, postprandial blood glucose, iAUC, or HbA1c levels. Based on the changes in the test group, three months of Mg^2+^ supplementation may not be sufficient; therefore, longer supplementation periods may be required. A meta-analysis of clinical trials revealed that oral supplementation of Mg^2+^ markedly improves the HOMA-IR index without impacting blood glucose, insulin, or HbA1c levels in patients with diabetes and individuals without diabetes. However, oral Mg^2+^ supplementation in the study subgroup with a supplementary period of four months or more shows notable improvement in fasting blood glucose concentration and HOMA-IR index compared to the subgroup with a supplementary period of under four months [18].

There was no positive effect on the HbA1c level following Mg^2+^ supplementation in this study, possibly because the HbA1c level is an index of total blood glucose concentration for the preceding 3–4 months [34]. Therefore, the HbA1c level may not apply to blood glucose control effects in short-period trials. Song et al. [35] presumed that 1769 participants were required per treatment group and that elemental Mg^2+^ should be administered at least 360 mg per day for at least four months to confirm the effects on HbA1c value.

The current study findings provide evidence that Mg^2+^ supplementation alleviates IR in individuals with pre-diabetes. Indices designed to assess insulin sensitivity using fasting specimens (HOMA-IR, QUICKI, and HOMA-β) and the OGTT-derived index (ISI_stumvoll_) improved after Mg^2+^ supplementation for three months. ISI_Stumvoll_ showed a marked improvement, whereas ISI_0,120_ did not. There may be several reasons for the heterogeneous response of the IR index. The ISI_0,120_ may be insensitive to detect insulin concentration changes, and longer durations of Mg^2+^ supplementation may be required to achieve remarkable results. It was hypothesized that Mg^2+^ primarily affects liver function rather than peripheral IR. Similarly, an index designed to assess insulin sensitivity using fasting samples that primarily reflects the fasting blood glucose level in the post-absorptive state and hepatic IR is an indicator of hepatic glucose production that is markedly improved by Mg^2+^ supplementation [36].

C-peptide is the cleavage product of proinsulin, the first molecule in the insulin synthesis pathway. C-peptide is stored in the secretory granules of the Golgi complex of pancreatic beta cells, removed from proinsulin, and secreted with insulin. Although C-peptide and insulin are secreted in the same molar quantity, the former has a longer half-life in blood and, thus, serves as an indicator of the pancreas’ insulin secretory function [37]. Additionally, unlike insulin, the liver cannot metabolize C-peptide secreted into the portal vein. Hence, measuring blood C-peptide levels can accurately measure portal vein insulin secretion and serve as an indicator of beta cell function [38]. In this study, insulin resistance was improved (Table 8), and C-peptide levels decreased (Table 7) in the Mg^2+^ intake group. This further confirms that Mg^2+^ intake improves insulin resistance and is expected to further improve the protective function of pancreatic beta cells.

Mg^2+^ is a critical component of glucose metabolism in pancreatic beta cells [39]; higher Mg^2+^ concentrations are associated with greater insulin sensitivity [40]. The influence of Mg^2+^ on insulin activity was proposed at the start of the 1980s [39]; subsequently, several clinical studies have observed the critical role of Mg^2+^ in insulin-related metabolism, suggesting that Mg^2+^ can be used to prevent T2D by alleviating IR [41].

Similar to the observations of this study, previous studies have shown the efficacy of Mg^2+^ in alleviating IR in individuals with pre-diabetes. Mg^2+^ supplementation in patients with hypomagnesemia improves insulin sensitivity and the function of beta cells of pancreatic islets of Langerhans [17,39,40]. The present study results are consistent with those of a previous clinical trial, demonstrating that treatment with 365 mg/day of Mg^2+^ for six months remarkably improved fasting insulin and IR in individuals without diabetes [42]. A study on American adults assessed the long-term effects of Mg^2+^ supplementation on insulin metabolism, reporting that increased Mg^2+^ intake lowers long-term IR when HOMA-IR is measured repeatedly over 20 years [43].

The results of this study confirmed a previously reported association between increased Mg^2+^ ingestion and reduced incidence of T2D [44,45]. The observation that ingesting high Mg^2+^ concentrations lowers the incidence of T2D is in line with previous findings [17,39,40]. Furthermore, previous clinical trials on Mg^2+^ supplementation in patients with diabetes and individuals without diabetes showed that Mg^2+^ supplements can improve blood glucose levels, insulin-related metabolism, and the function of beta cells of pancreatic islets of Langerhans [41,46,47].

Interestingly, the present study findings suggest that the link between Mg^2+^-related metabolic disorders is stronger when hyperinsulinemia and IR are included in the definition of metabolic disorders than when hyperglycemia or impaired blood glucose responses are included. Mg^2+^ ingestion is valuable for maintaining insulin metabolism and overall health since insulin levels and IR elevation are precursors of chronically high fasting glucose levels [48]. 

Hypomagnesemia is thought to have detrimental effects on beta-cell proliferation and mass, affecting insulin production and secretion [39,49]. High circulating insulin levels over a long period in IR can increase Mg^2+^ excretion in the kidney [38]. Conversely, Mg^2+^ substitution does not alleviate IR or improve metabolic regulation in patients with metabolic syndromes [50]. These results can be attributed to Mg^2+^ formulation, dose, and duration of administration. The supplement was administered for three months in the present study, whereas Mg^2+^ supplementation periods ranged from one to six months in previous studies [12,42,47,50,51], and better results were obtained in trials with prolonged supplementation periods.

However, a few studies show that Mg^2+^ does not affect glucose metabolism [52,53,54,55]. This may be because most studies have measured total magnesium in the bloodstream as free or bioavailable magnesium is not easy to measure. Ultimately, it is the amount of magnesium that is absorbed and utilized that is relevant.

Mg^2+^ occurs in various forms. Cooperman [56] suggested that more water-soluble magnesium is superior as it is relatively well absorbed, inexpensive, chemically stable, and does not cause diarrhea when taken in appropriate amounts. Magnesium oxide is used in most Mg^2+^ supplements and is cheaper than other forms; however, it is poorly absorbed and is more likely to cause diarrhea. However, magnesium chloride, the main ingredient in the clinical trial product in this study, can be absorbed better as it is in a more water-soluble form when ingested. In particular, magnesium chloride is less likely to cause diarrhea and is recommended at high doses, suggesting that the aqueous state is optimal for ingestion.

Mg^2+^ can alleviate IR through multiple mechanisms. First, Mg^2+^ can improve insulin secretion by pancreatic beta cells [39]. These findings on IR have been verified by previous studies demonstrating that Mg^2+^ can reduce oxidative stress [57], pro-inflammatory cytokine levels [58], and acute-phase protein content [59,60]. Second, Mg^2+^ directly affects tyrosine kinase activity and the autophosphorylation of the beta subunits of the insulin receptor and its downstream pathways [61,62,63]. Finally, Mg^2+^ plays a key role in muscle metabolism and mitigates IR in muscles [64]. Similarly, Mg^2+^ deficiency is involved in impaired glucose metabolism since it blocks the insulin pathway, reduces kinase dependence, and induces an acute-phase response related to a decrease in insulin sensitivity and subsequent glucose metabolism disorder [65].

Potassium (K^+^) is an essential mineral that plays a key role in determining the intracellular osmotic pressure and acid–base balance. K^+^ intake exhibits a blood pressure-lowering effect via the renin–angiotensin system-mediated sodium (Na^+^) excretion, in which renin secretion inhibition results in reduced Na^+^ reabsorption, more K^+^ is discharged, and blood pressure is lowered. In addition, as the K^+^ concentration increases, the activation of Na^+^–K^+^–ATPase pumps causes vascular expansion via hyperpolarization of vascular endothelial cells [66]. Epidemiologic studies suggest that increased K^+^ and Mg^2+^ intake and consumption of fruits and vegetables rich in antioxidant nutrients help lower the blood pressure [67,68]. Although the intervention used in this study contains K^+^ and Na^+^, it is unlikely that these mineral components are potential confounders. However, after improving the methods for mineral extraction from deep seawater, we will conduct clinical trials using Mg^2+^ alone as an intervention.

In this study, Mg^2+^ was set as a functional component in deep seawater mineral extract, and its ability to improve insulin resistance was verified. However, it is necessary to improve the current deep seawater mineral extraction method further and develop a separate extraction method for each mineral for mass production. Each component of the minerals extracted through this method as well as combinations of minerals related to medical efficacy must be evaluated to clearly establish the relationships between the mineral components and efficacy. Additionally, it is necessary to identify differences in medical efficacy through comparative verification between deep seawater minerals and general minerals.

This study has certain limitations. First, given the patient-directed nature of this study, it was not possible to ensure the prescribed amount of Mg^2+^ was consumed, impacting the accuracy of the results. Second, any change in the blood glucose and HbA1c levels could not be measured as the Mg^2+^ intake period was short (3 months). Accordingly, more meaningful results may be obtained if a longer Mg^2+^ intake period is employed in future clinical trials with more patients and changes in intake route and product formulations assessed. When preparing products for future clinical trials, we plan to analyze the minor components in the clinical trial product to interpret its effects and include a control group that receives Mg^2+^ supplementation alone.

## 5. Conclusions

This study supports the proposition that individuals with pre-diabetes can benefit from Mg^2+^ supplements to improve insulin metabolism and potentially lower the progression from pre-diabetes to diabetes to supplement lifestyle intervention programs. These results also support the hypothesis that Mg^2+^ supplementation markedly alleviates IR in individuals with pre-diabetes and may help inform the design of public health strategies focused on diabetes risk reduction.

## Figures and Tables

**Table 1:** Contents of deep seawater, deep seawater mineral extract (test product), and control product.

	Component	Deep Seawater Mineral Extract		Placebo	
		Content (mL)	Mixing Ratio (%)	Content (mL)	Mixing Ratio (%)
Main component	Deep seawater mineral extract	1.938	3.23	–	–
Minor component	Purified water	52.668	87.78	54.606	91.01
	Red grapefruit concentrate	3.600	6.00	3.600	6.00
	Prune concentrate	0.300	0.50	0.300	0.50
	Xanthan gum	0.090	0.15	0.090	0.15
	Sucralose	0.024	0.04	0.024	0.04
	Enzymatically treated stevia	0.060	0.10	0.060	0.10
	Anhydrous citric acid	0.600	1.00	0.600	1.00
	Grapefruit flavor	0.720	1.20	0.720	1.20
Total		60.000	100.00	60.000	100.00
Nutrient					
	Carbohydrate (g)	6.16	N/A	5.14	N/A
	Fat (g)	0.07	N/A	0.07	N/A
	Protein (g)	0.13	N/A	0.12	N/A
	Energy (Kcal)	25.82	N/A	21.69	N/A
Mineral/Property					
	Magnesium (Mg^2+^)	128.2	N/A	–	N/A
	Calcium (Ca^2+^)	–	N/A	–	N/A
	Sodium (Na^+^)	43.0	N/A	–	N/A
	Potassium (K^+^)	40.0	N/A	–	N/A
		**Concentrated deep seawater (mg/L)**			
	Magnesium (Mg^2+^)	2971.0	N/A		
	Calcium (Ca^2+^)	142.0	N/A		
	Sodium (Na^+^)	284.0	N/A		
	Potassium (K^+^)	–	N/A		
	Hardness	12,536.0 ^a^	N/A		

^a^ 2971.0 (mg/L) × 4.1 + 142.0 (mg/L) × 2.5.

**Table 2:** Demographic data.

	Control Group (*n* = 40)	Test Group (*n* = 40)	Total (*n* = 80)	*p*-Value
Sex (male/female)	11/29	14/26	25/55	0.4693
Age (years)	53.28 ± 9.28	55.80 ± 9.73	54.54 ± 9.53	0.2386
Height (cm)	162.48 ± 7.06	162.68 ± 7.55	162.58 ± 7.26	0.9029
Weight (kg)	68.74 ± 13.29	66.78 ± 11.59	67.76 ± 12.43	0.4842
BMI (kg/m^2^)	25.92 ± 4.08	25.13 ± 3.44	25.52 ± 3.77	0.3471
Current smoker (*n*, %)	3 (7.5)	4 (10.0)	7 (8.8)	0.7119
Amount of smoking, cigarette/day	10.00 ± 0.00	13.75 ± 4.79	12.14 ± 3.93	0.2152
Alcohol (*n*, %)	24 (60.0)	20 (50)	44 (55.0)	0.3687
Alcohol consumption (g/week)	66.33 ± 124.75	48.60 ± 56.94	58.27 ± 99.18	0.5376
Hypertension (*n*, %)	7 (17.5)	6 (15.0)	13 (16.3)	0.7618
FPG (mg/dL)	100.10 ± 9.24	99.10 ± 7.41	99.60 ± 8.33	0.5947
2h-PPG (mg/dL)	166.40 ± 16.41	161.95 ± 18.55	164.18 ± 17.54	0.2593
HbA1c (%)	5.71 ± 0.32	5.77 ± 0.26	5.74 ± 0.29	0.3427
Mg (mg/dL)	2.12 ± 0.14	2.12 ± 0.17	2.12 ± 0.17	0.8954

BMI, body mass index; FPG, fasting plasma glucose; 2h-PPG, 2 h postprandial plasma glucose; HbA1c, glycosylated hemoglobin type A1c. Data are presented as the mean ± SD or numbers (percentage). The chi-square test, Fisher’s exact test, or independent *t*-test was used for the homogeneity test between the groups and the baseline homogeneity test.

**Table 3:** Patient compliance.

	Control Group (*n* = 37)	Test Group (*n* = 34)	Total (*n* = 71)	*p*-Value
Number of products to consume (bottles)	168.86 ± 8.70	167.29 ± 8.48	168.11 ± 8.57	0.4445
Number of products consumed (bottles)	160.57 ± 12.67	157.85 ± 10.85	159.27 ± 11.83	0.3378
Compliance (%)	95.09 ± 5.65	94.40 ± 5.25	94.73 ± 5.43	0.5993

Data are expressed as mean ± SD. Statistical analysis was conducted using the independent *t*-test.

**Table 4:** Change in dietary intake.

		Control Group (*n* = 37)	Test Group (*n* = 34)	*p*-Value ^a^
Total calories (kcal)	Baseline	1406.14 ± 528.73	1407.11 ± 444.73	0.9934
	6 weeks	1377.02 ± 453.99	1508.22 ± 446.23	
	Variation from baseline	−29.12 ± 548.48	101.12 ± 612.10	
	*p*-value ^b^	0.7486	0.3424	0.3477
	12 weeks	1473.19 ± 389.88	1456.66 ± 458.19	
	Variation from baseline	67.05 ± 521.45	49.55 ± 588.36	
	*p*-value ^b^	0.4392	0.6266	0.8947
Carbohydrate (g)	Baseline	206.78 ± 85.72	206.80 ± 68.86	0.9989
	6 weeks	204.41 ± 64.69	217.89 ± 66.10	
	Variation from baseline	−2.36 ± 81.71	11.09 ± 93.68	
	*p*-value ^b^	0.8613	0.4949	0.5203
	12 weeks	207.84 ± 65.00	221.57 ± 71.98	
	Change from baseline	1.06 ± 71.12	14.77 ± 96.31	
	*p*-value ^b^	0.9280	0.3778	0.4952
Fat (g)	Baseline	37.75 ± 23.10	37.18 ± 17.55	0.9084
	6 weeks	38.26 ± 27.06	43.17 ± 26.65	
	Variation from baseline	0.51 ± 27.71	5.99 ± 30.62	
	*p*-value ^b^	0.9108	0.2626	0.4320
	12 weeks	43.41 ± 20.69	38.64 ± 19.40	
	Variation from baseline	5.66 ± 27.15	1.46 ± 23.20	
	*p*-value ^b^	0.2126	0.7157	0.4876
Protein (g)	Baseline	60.36 ± 30.19	63.06 ± 29.16	0.7031
	6 weeks	55.54 ± 24.46	65.99 ± 26.42	
	Variation from baseline	−4.82 ± 32.38	2.92 ± 39.65	
	*p*-value ^b^	0.3708	0.6701	0.3687
	12 weeks	63.13 ± 22.07	60.25 ± 23.24	
	Variation from baseline	2.77 ± 33.50	−2.81 ± 36.43	
	*p*-value ^b^	0.6185	0.6556	0.5037
Dietary fiber (g)	Baseline	17.31 ± 8.16	19.80 ± 9.07	0.2273
	6 weeks	17.86 ± 8.55	19.83 ± 7.04	
	Variation from baseline	0.55 ± 8.61	0.02 ± 10.10	
	*p*-value ^b^	0.7006	0.9900	0.8134
	12 weeks	18.11 ± 7.42	20.34 ± 9.25	
	Variation from baseline	0.80 ± 8.07	0.54 ± 11.16	
	*p*-value ^b^	0.5515	0.7796	0.9112

Data are expressed as mean ± SD. ^a^ Compared between groups: *p*-value using the Wilcoxon rank-sum test. ^b^ Compared within groups: *p*-value using the Wilcoxon signed-rank test.

**Table 5:** Changes in physical activity.

		Control Group (*n* = 37)	Test Group (*n* = 34)	*p*-Value ^a^
Physical activity MET-min/week	Baseline	960 (360–1600)	1450 (240–3840)	0.2672
	6 weeks	960 (560–2160)	980 (240–2880)	
	Variation from baseline	0 (−600 to –1200)	0 (−960 to –740)	
	*p*-value ^b^	0.6083	0.9410	0.9447
	12 weeks	800 (0–1680)	1160 (120–2400)	
	Variation from baseline	0 (−1200 to −480)	0 (−1200 to –480)	
	*p*-value ^b^	0.2683	0.9329	0.5489

MET, metabolic equivalent of task. Data are expressed as median (interquartile range). ^a^ Compared between groups: *p*-value using the Wilcoxon rank-sum test. ^b^ Compared within groups: *p*-value using the Wilcoxon signed-rank test.

**Table 6:** Changes in the blood glucose levels compared to the baseline levels in the 75 g OGTT.

		Control Group (*n* = 38)	Test Group (*n* = 37)	*p*-Value ^a^
FPG (mg/dL)	Baseline	100.45 ± 9.35	98.95 ± 7.59	0.4482
	6 weeks	96.61 ± 9.92	98.30 ± 8.41	
	Variation from baseline	−3.84 ± 8.79	−0.65 ± 6.12	
	*p*-value ^b^	0.0105	0.5235	0.0718
	12 weeks	100.39 ± 9.78	98.95 ± 7.78	
	Variation from baseline	−0.05 ± 8.04	0.00 ± 5.57	
	*p*-value ^b^	0.9680	>0.9999	0.9738
PPG_0.5h_ (mg/dL)	Baseline	174.34 ± 26.67	173.46 ± 22.45	0.8774
	6 weeks	169.39 ± 26.56	172.78 ± 29.25	
	Variation from baseline	−4.95 ± 30.83	−0.68 ± 28.04	
	*p*-value ^b^	0.3290	0.8843	0.5324
	12 weeks	174.16 ± 23.55	168.24 ± 25.25	
	Variation from baseline	−0.18 ± 26.81	−5.22 ± 22.14	
	*p*-value ^b^	0.9664	0.1604	0.3791
PPG_1.0h_ (mg/dL)	Baseline	195.84 ± 39.74	198.30 ± 29.35	0.7622
	6 weeks	190.71 ± 41.05	191.05 ± 37.57	
	Variation from baseline	−5.13 ± 34.45	−7.24 ± 39.06	
	*p*-value ^b^	0.3645	0.2668	0.8045
	12 weeks	201.26 ± 35.59	198.84 ± 34.97	
	Variation from baseline	5.42 ± 28.01	0.54 ± 35.43	
	*p*-value ^b^	0.2404	0.9266	0.5095
PPG_1.5h_ (mg/dL)	Baseline	183.24 ± 29.23	181.43 ± 27.94	0.7855
	6 weeks	188.08 ± 36.30	179.73 ± 36.91	
	Variation from baseline	4.84 ± 32.25	−1.70 ± 39.14	
	*p*-value ^b^	0.3607	0.7928	0.4314
	12 weeks	186.08 ± 36.55	183.95 ± 39.05	
	Variation from baseline	2.84 ± 29.33	2.51 ± 36.91	
	*p*-value ^b^	0.5540	0.6812	0.9660
PPG_2.0h_ (mg/dL)	Baseline	166.39 ± 16.16	162.54 ± 18.58	0.3406
	6 weeks	164.55 ± 34.45	160.46 ± 33.22	
	Variation from baseline	−1.84 ± 33.81	−2.08 ± 36.36	
	*p*-value ^b^	0.7388	0.7297	0.9766
	12 weeks	165.95 ± 34.51	164.05 ± 31.92	
	Variation from baseline	−0.45 ± 30.97	1.51 ± 32.20	
	*p*-value ^b^	0.9295	0.7766	0.7889
Glucose iAUC_0–2h_ (h × mg/dL)	Baseline	143.01 ± 38.82	144.07 ± 32.64	0.8984
	6 weeks	146.17 ± 40.90	140.12 ± 43.92	
	Variation from baseline	3.16 ± 37.55	−3.96 ± 47.92	
	*p*-value ^b^	0.6067	0.6184	0.4754
	12 weeks	146.55 ± 39.10	143.42 ± 42.96	
	Variation from baseline	3.53 ± 31.50	−0.65 ± 39.09	
	*p*-value ^b^	0.4935	0.9196	0.6105

FPG, fasting plasma glucose; PPG, postprandial plasma glucose; iAUC, incremental area under the curve; OGTT, oral glucose tolerance test. Values are expressed as the mean ± SD. ^a^ Compared between groups; *p*-value using the independent *t*-test. ^b^ Compared within groups; *p*-value using the paired *t*-test.

**Table 7:** Fasting insulin, C-peptide, and insulin levels in the 75 g OGTT.

		Control Group (*n* = 38)	Test Group (*n* = 37)	*p*-Value ^a^
Fasting insulin (μU/mL)	Baseline	9.61 ± 5.08	10.79 ± 6.84	0.3990
	6 weeks	8.47 ± 4.65	9.78 ± 6.42	
	Variation from baseline	−1.14 ± 3.71	−1.01 ± 6.84	
	*p*-value ^b^	0.0660	0.3737	0.9217
	12 weeks	9.39 ± 6.00	8.32 ± 4.61	
	Variation from baseline	−0.22 ± 3.93	−2.47 ± 4.51	
	*p*-value ^b^	0.7338	0.0020	0.0238 *
Insulin_0.5h_ (μU/mL)	Baseline	64.62 ± 48.85	54.64 ± 28.16	0.2813
	6 weeks	66.89 ± 49.63	60.00 ± 40.36	
	Variation from baseline	2.28 ± 46.13	5.36 ± 23.44	
	*p*-value ^b^	0.7627	0.1729	0.7155
	12 weeks	62.87 ± 43.97	48.12 ± 28.22	
	Variation from baseline	−1.75 ± 29.03	−6.52 ± 22.46	
	*p*-value ^b^	0.7127	0.0859	0.4291
Insulin_1.0h_ (μU/mL)	Baseline	80.28 ± 54.17	79.18 ± 37.43	0.9184
	6 weeks	73.63 ± 48.30	78.15 ± 43.48	
	Variation from baseline	−6.64 ± 47.56	−1.02 ± 30.44	
	*p*-value ^b^	0.3946	0.8394	0.5431
	12 weeks	85.79 ± 52.30	69.41 ± 33.69	
	Variation from baseline	5.51 ± 44.84	−9.76 ± 31.37	
	*p*-value ^b^	0.4537	0.0663	0.0914
Insulin_1.5h_ (μU/mL)	Baseline	91.47 ± 59.58	86.07 ± 41.22	0.6488
	6 weeks	88.04 ± 54.72	82.08 ± 45.26	
	Variation from baseline	−3.43 ± 48.80	−3.99 ± 43.07	
	*p*-value ^b^	0.6676	0.5767	0.9579
	12 weeks	86.88 ± 49.21	83.37 ± 51.60	
	Variation from baseline	−4.59 ± 33.55	−2.69 ± 48.31	
	*p*-value ^b^	0.4042	0.7364	0.8444
Insulin_2.0h_ (μU/mL)	Baseline	93.26 ± 58.37	86.51 ± 43.90	0.5739
	6 weeks	89.36 ± 62.04	82.30 ± 42.84	
	Variation from baseline	−3.90 ± 56.18	−4.21 ± 50.58	
	*p*-value ^b^	0.6714	0.6155	0.9797
	12 weeks	90.44 ± 56.11	87.30 ± 57.22	
	Variation from baseline	−2.82 ± 35.18	0.79 ± 41.54	
	*p*-value ^b^	0.6244	0.9086	0.6857
C-peptide (ng/mL)	Baseline	2.29 ± 0.76	2.26 ± 0.75	0.8578
	6 weeks	2.40 ± 0.92	2.21 ± 0.59	
	Variation from baseline	0.10 ± 0.56	−0.06 ± 0.52	
	*p*-value ^b^	0.2677	0.5152	0.2087
	12 weeks	2.51 ± 0.89	2.15 ± 0.67	
	Variation from baseline	0.21 ± 0.70	−0.12 ± 0.51	
	*p*-value ^b^	0.0666	0.1715	0.0220 *
IGI	Baseline	0.80 ± 0.67	0.60 ± 0.29	0.1006
	6 weeks	0.84 ± 0.63	0.67 ± 0.48	
	Variation from baseline	0.04 ± 0.67	0.07 ± 0.32	
	*p*-value ^b^	0.7183	0.1788	0.7891
	12 weeks	0.72 ± 0.52	0.58 ± 0.38	
	Variation from baseline	−0.07 ± 0.56	−0.02 ± 0.27	
	*p*-value ^b^	0.4253	0.6567	0.5977

OGTT, oral glucose tolerance test. IGI, insulinogenic index. Data are expressed as the mean ± SD. * *p* < 0.05. ^a^ Comparison between groups; *p*-value determined using the independent *t*-test. ^b^ Comparison within groups; *p*-value determined using the paired *t*-test.

**Table 8:** Insulin sensitivity surrogate markers.

		Control Group (*n* = 38)	Test Group (*n* = 37)	*p*-Value ^a^
HOMA-IR	Baseline	2.39 ± 1.26	2.70 ± 1.82	0.3947
	6 weeks	2.81 ± 2.39	2.33 ± 1.10	
	Variation from baseline	0.42 ± 1.72	−0.37 ± 1.40	
	*p*-value ^b^	0.1375	0.1152	0.0316 *
	12 weeks	2.98 ± 1.80	2.41 ± 1.48	
	Variation from baseline	0.59 ± 1.48	−0.29 ± 1.40	
	*p*-value ^b^	0.0185	0.2214	0.0102 *
HOMA-β (%)	Baseline	97.43 ± 57.00	106.15 ± 58.71	0.5159
	6 weeks	106.91 ± 64.56	93.29 ± 36.69	
	Variation from baseline	9.48 ± 56.54	−12.86 ± 49.72	
	*p*-value ^b^	0.3080	0.1244	0.0736
	12 weeks	110.67 ± 64.19	90.54 ± 37.70	
	Variation from baseline	13.24 ± 50.52	−15.61 ± 44.62	
	*p*-value ^b^	0.1148	0.0402	0.0107 *
QUICKI	Baseline	0.35 ± 0.03	0.34 ± 0.04	0.8500
	6 weeks	0.34 ± 0.03	0.34 ± 0.03	
	Variation from baseline	0.00 ± 0.02	0.00 ± 0.02	
	*p*-value ^b^	0.1782	0.9472	0.3492
	12 weeks	0.33 ± 0.03	0.35 ± 0.04	
	Variation from baseline	−0.01 ± 0.03	0.00 ± 0.03	
	*p*-value ^b^	0.0188	0.5573	0.0378 *
ISI_stumvoll_	Baseline	0.108 ± 0.015	0.109 ± 0.013	0.7540
	6 weeks	0.108 ± 0.015	0.110 ± 0.013	
	Variation from baseline	0.000 ± 0.003	0.001 ± 0.004	
	*p*-value ^b^	0.5751	0.3255	0.6756
	12 weeks	0.108 ± 0.015	0.111 ± 0.013	
	Variation from baseline	0.000 ± 0.003	0.002 ± 0.003	
	*p*-value ^b^	0.9462	0.0002	0.0073 **
ISI_0,120_	Baseline	34.95 ± 8.10	35.38 ± 7.35	0.8127
	6 weeks	35.62 ± 8.46	41.90 ± 37.24	
	Variation from baseline	0.67 ± 7.55	6.52 ± 36.53	
	*p*-value ^b^	0.5876	0.2848	0.3457
	12 weeks	35.05 ± 9.83	41.43 ± 37.01	
	Variation from baseline	0.10 ± 6.33	6.05 ± 36.06	
	*p*-value ^b^	0.9231	0.3139	0.3285
HbA1c (%)	Baseline	5.71 ± 0.32	5.78 ± 0.27	0.3485
	12 weeks	5.68 ± 0.29	5.73 ± 0.30	
	Variation from baseline	−0.03 ± 0.16	−0.05 ± 0.14	
	*p*-value ^b^	0.2646	0.0481	0.6195

HOMA-IR, homeostatic model assessment for insulin resistance; HOMA-β, homeostatic model assessment of β-cell function; QUICKI, quantitative insulin sensitivity check index; ISI, insulin sensitivity index. Values are expressed as the mean ± SD * *p* < 0.05. ** *p* < 0.01. ^a^ Comparison between groups: *p*-value was determined using the independent *t*-test. ^b^ Comparison within groups: *p*-value was determined using the paired *t*-test.

## Data Availability

The original contributions presented in the study are included in the article; further inquiries can be directed to the corresponding author.

## Supplementary Materials

The following supporting information can be downloaded at: https://www.mdpi.com/article/10.3390/medicina60081265/s1. Figure S1: CONSORT 2010 flow diagram; Table S1: Criteria for exclusion of study participants; Table S2: CONSORT 2010 checklist of information to include when reporting a randomized trial; Table S3: Schedule summary; Table S4-1: Changes in the blood glucose levels compared to the baseline levels in the 75 g OGTT in 30–40-year-olds; Table S4-2: Changes in the blood glucose levels compared to the baseline levels in the 75 g OGTT in 50–59-year-olds; Table S4-3: Changes in the blood glucose levels compared to the baseline levels in the 75 g OGTT in 60–69-year-olds; Table S5-1: Fasting insulin, C-peptide, and insulin levels in the 75 g OGTT in 30–40-year-olds; Table S5-2: Fasting insulin, C-peptide, and insulin levels in the 75 g OGTT in 50–59-year-olds; Table S5-3: Fasting insulin, C-peptide, and insulin levels in the 75 g OGTT in 60–69-year-olds; Table S6-1: Insulin sensitivity surrogate markers in 30–40-year-olds; Table S6-2: Insulin sensitivity surrogate markers in 50–59-year-olds; Table S6-3: Insulin sensitivity surrogate markers in 60–69-year-olds.
