# Supplementary material for: Magnesium from Deep Seawater as a Potentially Effective Natural Product against Insulin Resistance: A Randomized Trial
Source: Medicina (Kaunas). 2024 Aug 5;60(8):1265. doi: 10.3390/medicina60081265 (PMC11355969; doi:10.3390/medicina60081265)
Supplement: Supplementary file 1 [file medicina-60-01265-s001.zip › medicina-3043914-supplementary.pdf]

Supplementary Material

Magnesium from Deep Seawater as a Potentially Effective Natural Product against Insulin Resistance: A Randomized Trial

Ji Yeon Ham [1], You Kyung Jang [2], Byong Yeob Jeon [2] and Yun Hee Shon [3,*]

[1] Department of Laboratory Medicine, Kyungpook National University Chilgok Hospital, School of Medicine, Kyungpook National University, 807 Hogukro Buk-gu, Daegu 41404, Republic of Korea
[2] QBM Research Institute, QBM Co., Ltd., 7-25 Gangnam-daero 27-gil, Seocho-gu, Seoul 06752, Republic of Korea
[3] Bio-Medical Research Institute, Kyungpook National University Hospital, 135 Dongdukro Jung-gu, Daegu 41940, Republic of Korea
* Correspondence: yhshon@knuh.kr; Tel.: +82-53-200-6952

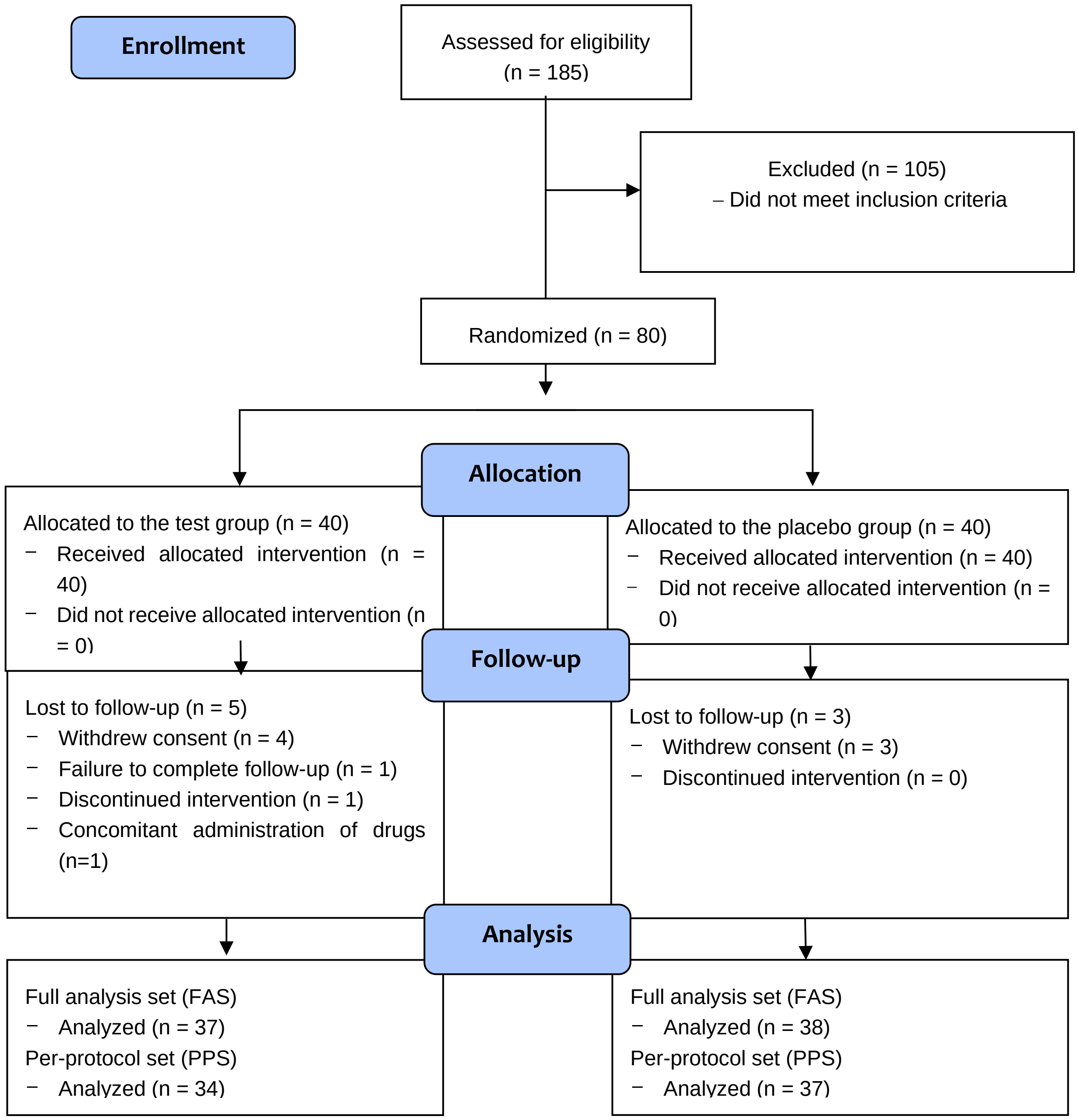

Figure S1. CONSORT 2010 flow diagram.

Table S1. Criteria for exclusion of study participants.

Subjects enrolled in this clinical trial did not fall under any of the following categories:	
1	A fasting blood glucose of ≥ 140 mg/dL
2	Glycated hemoglobin (HbA1c) level of ≥ 7.0% in the screening test
3	Diagnosed with diabetes and undergoing treatment
4	Weight loss of 10% within the last three months
5	Systolic blood pressure of 160 mmHg or diastolic blood pressure of ≥ 100 mmHg (Note: those with stably controlled blood pressure with medication were permitted)
6	Consumed systemic corticosteroids within four weeks before screening
7	Had taken medications such as hypoglycemic drugs, anti-obesity drugs, or lipid-lowering drugs within three months before screening; consumed functional health foods designed to regulate blood glucose, combat obesity, or improve lipid profiles, or any medications or health supplements that might influence the interpretation of the results of this study within two months of the screening test
8	Consumed mineral supplements within three months before screening
9	History of hepatobiliary conditions, kidney disorders, nervous system (central or peripheral) disorders, respiratory system conditions (e.g., asthma), endocrine system disorders (e.g., thyroid disease), cardiovascular system ailments (e.g., congestive heart failure, coronary artery disease, and myocardial infarction), blood-related tumors, urinary system disorders, mental health issues, musculoskeletal system disorders, immune system disorders (e.g., rheumatoid arthritis, systemic lupus erythematosus, and immunodeficiency diseases), or clinically significant illnesses as determined by the investigator, rendering them ineligible for participation in the study
10	Alcoholism, drug abuse, or dependency
11	History of potential gastrointestinal diseases, such as Crohn's disease, or had undergone gastrointestinal surgery (except for simple procedures such as appendectomy or hernia surgery) that could potentially impact the absorption of the test product
12	History of hypersensitivity or clinically significant allergic reactions to drugs and clinical trial products (e.g., magnesium)
13	Taken part in another clinical trial within three months prior to the screening test
14	Laboratory test results indicating AST and ALT levels three times higher than the upper limit of the reference value range or serum creatinine levels exceeding 2.0 mg/dL

15	Females of childbearing age who did not consent to the use of an effective contraceptive method during this clinical trial
16	Pregnant or lactating females
17	Participants deemed unfit to participate in the clinical trial by the study director, based on diagnostic test results or other relevant reasons

Table S2. CONSORT 2010 checklist of information to include when reporting a randomized trial.

Section/Topic	Item No	Checklist item	Reported on page no.
Title and abstract			
	1a	Identification as a randomized trial in the title	1
	1b	Structured summary of trial design, methods, results, and conclusions (for specific guidance see CONSORT for abstracts)	1
Introduction			
Background and objectives	2a	Scientific background and explanation of rationale	1–2
	2b	Specific objectives or hypotheses	2
Methods			
Trial design	3a	Description of trial design (such as parallel, factorial) including allocation ratio	4
	3b	Important changes to methods after trial commencement (such as eligibility criteria), with reasons	N/A
Participants	4a	Eligibility criteria for participants	3
	4b	Settings and locations from where the data were collected	2–3
Interventions	5	The interventions for each group with sufficient details to allow replication, including how and when they were actually administered	3–5
Outcomes	6a	Completely defined pre-specified primary and secondary outcome measures, including how and when they were assessed	3–5
	6b	Any changes to trial outcomes after the trial commenced, with reasons	N/A
Sample size	7a	How sample size was determined	3,
	7b	When applicable, explanation of any interim analyses and stopping guidelines	N/A
Randomization:			

Sequence generation	8a	Method used to generate the random allocation sequence	5
	8b	Type of randomization; details of any restriction (such as blocking and block size)	4
Allocation concealment mechanism	9	Mechanism used to implement the random allocation sequence (such as sequentially numbered containers), describing any steps taken to conceal the sequence until interventions were assigned	5
Implementation	10	Who generated the random allocation sequence, who enrolled participants, and who assigned participants to interventions	4
Blinding	11a	If done, who was blinded after assignment to interventions (for example, participants, care providers, those assessing outcomes) and how	4
	11b	If relevant, description of the similarity of interventions	5
Statistical methods	12a	Statistical methods used to compare groups for primary and secondary outcomes	5–6
	12b	Methods for additional analyses, such as subgroup analyses and adjusted analyses	5–6
Results			
Participant flow (a diagram is strongly recommended)	13a	For each group, the numbers of participants who were randomly assigned, received intended treatment, and were analyzed for the primary outcome	6–7
	13b	For each group, losses and exclusions after randomization, together with reasons	6–7
Recruitment	14a	Dates defining the periods of recruitment and follow-up	6–7
	14b	Why the trial ended or was stopped	6–7
Baseline data	15	A table showing baseline demographic and clinical characteristics for each group	6–7

Numbers analyzed	16	For each group, number of participants (denominator) included in each analysis and whether the analysis was by original assigned groups	7
Outcomes and estimation	17a	For each primary and secondary outcome, results for each group, and the estimated effect size and its precision (such as 95% confidence interval)	7–12
	17b	For binary outcomes, presentation of both absolute and relative effect sizes is recommended	7–12
Ancillary analyses	18	Results of any other analyses performed, including subgroup analyses and adjusted analyses, distinguishing pre-specified from exploratory	7–12
Harms	19	All important harms or unintended effects in each group (for specific guidance see CONSORT for harms)	N/A
Discussion			
Limitations	20	Trial limitations, addressing sources of potential bias, imprecision, and, if relevant, multiplicity of analyses	14
Generalizability	21	generalizability (external validity, applicability) of the trial findings	12–13
Interpretation	22	Interpretation consistent with results, balancing benefits and harms, and considering other relevant evidence	14
Other information			
Registration	23	Registration number and name of trial registry	3
Protocol	24	Where the full trial protocol can be accessed, if available	N/A
Funding	25	Sources of funding and other support (such as supply of drugs), role of funders	15

Table S3. Schedule summary.

Schedule		Screening	First Visit	Second Visit	Third Visit
**Items**		**D-14 to D-1**	**D0**	**D42 (± 7)**	**D84 (± 7)**
Written consent		●			
Demographic information/medical history		●			
Drug administration investigation		●	●	●	●
Drinking · smoking history survey		●		●	●
Investigation of changes in medical conditions			●		
Physical examination		●			●
Vital signs		●	●	●	●
Laboratory tests		●			●
Pregnancy test (urine hCG)		●			●
Electrocardiogram		●			●
Body measurement		●	●		●
Confirm selection/exclusion criteria		●	●		
Randomization			●		
Efficacy evaluation	75 g-OGTT (blood glucose, insulin)	●		●	●
	C-peptide	●		●	●
	Glycosylated hemoglobin	●			●
	hs-CRP	●		●	●
Sample collection for further analysis		●			●
Provision of products for clinical trial			●	●	
Dietary intake survey		●		●	●
Physical activity survey		●		●	●
Collection of returned products, compliance evaluation				●	●
Adverse reaction survey				●	●

hCG, human chorionic gonadotropin; OGTT, oral glucose tolerance test; hs-CRP, high-sensitivity C-reactive protein

Table S4-1. Changes in the blood glucose levels compared to the baseline levels in the 75 g OGTT in 30–40-year-olds.

		Control group (n = 14)	Test group (n = 10)	*P*-value[a]
FPG (mg/dL)	Baseline	97.71 ± 7.05	102.10 ± 10.59	0.2350
	6 weeks	92.36 ± 8.79	100.30 ± 7.24	
	Variation from baseline	-5.36 ± 9.22	-1.80 ± 6.89	
	P-value[b]	0.0488	0.4303	0.3145
	12 weeks	97.21 ± 8.89	102.20 ± 9.75	
	Variation from baseline	-0.50 ± 7.12	0.10 ± 7.22	
	P-value[b]	0.7969	0.9660	0.8415
$PPG_{0.5h}$ (mg/dL)	Baseline	170.57 ± 30.24	176.50 ± 24.10	0.6128
	6 weeks	160.71 ± 24.80	180.40 ± 23.59	
	Variation from baseline	-9.86 ± 35.77	3.90 ± 21.57	
	P-value[b]	0.3214	0.5815	0.2919
	12 weeks	167.57 ± 27.62	175.60 ± 21.06	
	Variation from baseline	-3.00 ± 22.77	-0.90 ± 19.18	
	P-value[b]	0.6303	0.8853	0.8146
$PPG_{1.0h}$ (mg/dL)	Baseline	187.21 ± 37.31	193.90 ± 34.46	0.6596
	6 weeks	173.57 ± 35.68	195.70 ± 34.23	
	Variation from baseline	-13.64 ± 45.78	1.80 ± 30.26	
	P-value[b]	0.2850	0.8550	0.3632
	12 weeks	192.57 ± 35.99	199.50 ± 40.47	
	Variation from baseline	5.36 ± 33.98	5.60 ± 28.91	
	P-value[b]	0.5654	0.5553	0.9855
$PPG_{1.5h}$ (mg/dL)	Baseline	173.79 ± 30.79	184.60 ± 28.69	0.3925
	6 weeks	171.14 ± 34.41	188.40 ± 26.80	
	Variation from baseline	-2.64 ± 39.12	3.80 ± 29.59	
	P-value[b]	0.8044	0.6941	0.6657
	12 weeks	174.43 ± 29.06	180.10 ± 43.36	
	Variation from baseline	0.64 ± 25.00	-4.50 ± 37.85	
	P-value[b]	0.9248	0.7156	0.6916
$PPG_{2.0h}$ (mg/dL)	Baseline	163.21 ± 17.98	171.00 ± 25.60	0.3896
	6 weeks	150.79 ± 31.67	156.50 ± 29.04	
	Variation from baseline	-12.43 ± 33.51	-14.50 ± 33.30	
	P-value[b]	0.1885	0.2018	0.8824
	12 weeks	151.79 ± 23.50	152.80 ± 40.84	
	Variation from baseline	-11.43 ± 22.41	-18.20 ± 37.26	
	P-value[b]	0.0788	0.1568	0.5837
Glucose $iAUC_{0-2h}$ (h*mg/dL)	Baseline	135.66 ± 41.24	141.58 ± 34.03	0.7137
	6 weeks	128.80 ± 41.46	145.85 ± 27.71	

Variation from baseline	-6.86 ± 49.29	4.28 ± 25.11	
P-value[b]	0.6112	0.6033	0.4773
12 weeks	135.11 ± 38.49	136.95 ± 40.39	
Variation from baseline	-0.55 ± 37.52	-4.63 ± 27.77	
P-value[b]	0.9571	0.6111	0.7741

FPG, fasting plasma glucose; PPG, postprandial plasma glucose; iAUC, incremental area under the curve; OGTT, oral glucose tolerance test. Values are expressed as the mean ± SD. [a] Compared between groups; *P*-value using the independent *t*-test. [b] Compared within groups; *P*-value using the paired *t*-test.

Table S4-2. Changes in the blood glucose levels compared to the baseline levels in the 75 g OGTT in 50–59-year-olds.

		Control group (n = 12)	Test group (n = 12)	*P*-value[a]
FPG (mg/dL)	Baseline	101.75 ± 12.35	100.00 ± 5.36	0.6590
	6 weeks	100.92 ± 8.85	102.58 ± 8.32	
	Variation from baseline	-0.83 ± 8.24	2.58 ± 5.58	
	P-value[b]	0.7328	0.1373	0.2472
	12 weeks	102.92 ± 7.66	100.25 ± 5.07	
	Variation from baseline	1.17 ± 10.21	0.25 ± 3.19	
	P-value[b]	0.6997	0.7913	0.7712
$PPG_{0.5h}$ (mg/dL)	Baseline	179.08 ± 19.04	172.75 ± 22.89	0.4690
	6 weeks	179.33 ± 17.71	175.75 ± 33.92	
	Variation from baseline	0.25 ± 26.76	3.00 ± 32.08	
	P-value[b]	0.9748	0.7520	0.8217
	12 weeks	179.25 ± 18.67	155.75 ± 27.66	
	Variation from baseline	0.17 ± 31.16	-17.00 ± 23.06	
	P-value[b]	0.9855	0.0268	0.1392
$PPG_{1.0h}$ (mg/dL)	Baseline	192.75 ± 36.76	189.67 ± 27.41	0.8180
	6 weeks	195.42 ± 34.70	187.25 ± 46.31	
	Variation from baseline	2.67 ± 24.60	-2.42 ± 49.44	
	P-value[b]	0.7145	0.8686	0.7539
	12 weeks	194.58 ± 35.76	189.42 ± 42.32	
	Variation from baseline	1.83 ± 21.54	-0.25 ± 48.20	
	P-value[b]	0.7736	0.9860	0.8931
$PPG_{1.5h}$ (mg/dL)	Baseline	179.67 ± 26.25	172.42 ± 32.03	0.5504
	6 weeks	196.25 ± 37.11	170.67 ± 49.66	
	Variation from baseline	16.58 ± 27.39	-1.75 ± 44.10	
	P-value[b]	0.0599	0.8931	0.2341
	12 weeks	184.92 ± 41.62	183.00 ± 45.92	
	Variation from baseline	5.25 ± 36.16	10.58 ± 50.98	
	P-value[b]	0.6249	0.4871	0.7703
$PPG_{2.0h}$ (mg/dL)	Baseline	164.92 ± 15.42	157.25 ± 13.94	0.2148
	6 weeks	172.00 ± 41.24	160.92 ± 42.67	
	Variation from baseline	7.08 ± 40.33	3.67 ± 42.28	
	P-value[b]	0.5553	0.7694	0.8413
	12 weeks	173.08 ± 39.29	162.08 ± 34.24	
	Variation from baseline	8.17 ± 34.95	4.83 ± 32.15	
	P-value[b]	0.4354	0.6129	0.8101
Glucose $iAUC_{0-2h}$ (h*mg/dL)	Baseline	138.92 ± 32.87	131.73 ± 33.11	0.5989
	6 weeks	151.90 ± 34.83	128.27 ± 59.13	

Variation from baseline	12.98 ± 28.03	-3.46 ± 62.50	
P-value[b]	0.1370	0.8516	0.4187
12 weeks	142.54 ± 43.60	129.32 ± 55.57	
Variation from baseline	3.63 ± 33.62	-2.41 ± 60.32	
P-value[b]	0.7159	0.8924	0.7649

FPG, fasting plasma glucose; PPG, postprandial plasma glucose; iAUC, incremental area under the curve; OGTT, oral glucose tolerance test. Values are expressed as the mean ± SD. [a] Compared between groups; *P*-value using the independent *t*-test. [b] Compared within groups; *P*-value using the paired *t*-test.

Table S4-3. Changes in the blood glucose levels compared to the baseline levels in the 75 g OGTT in 60–69-year-olds.

		Control group (n = 12)	Test group (n = 15)	*P*-value[a]
FPG (mg/dL)	Baseline	102.33 ± 8.19	96.00 ± 6.01	**0.0289**[†]
	6 weeks	97.25 ± 10.86	93.53 ± 7.14	
	Variation from baseline	-5.08 ± 8.77	-2.47 ± 5.29	0.3463 (0.6432[†])
	P-value[b]	0.0700	0.0925	
	12 weeks	101.58 ± 12.18	95.73 ± 7.36	
	Variation from baseline	-0.75 ± 7.14	-0.27 ± 6.17	0.8518 (0.8326[†])
	P-value[b]	0.7227	0.8695	
$PPG_{0.5h}$ (mg/dL)	Baseline	174.00 ± 30.10	172.00 ± 22.40	0.8446
	6 weeks	169.58 ± 33.59	165.33 ± 28.80	
	Variation from baseline	-4.42 ± 30.14	-6.67 ± 29.13	
	P-value[b]	0.6217	0.3903	0.8459
	12 weeks	176.75 ± 22.90	173.33 ± 23.51	
	Variation from baseline	2.75 ± 28.55	1.33 ± 20.68	
	P-value[b]	0.7449	0.8064	0.8823
$PPG_{1.0h}$ (mg/dL)	Baseline	209.00 ± 44.97	208.13 ± 26.08	0.9504
	6 weeks	206.00 ± 48.03	191.00 ± 34.10	
	Variation from baseline	-3.00 ± 27.32	-17.13 ± 35.09	
	P-value[b]	0.7109	0.0795	0.2635
	12 weeks	218.08 ± 31.58	205.93 ± 23.54	
	Variation from baseline	9.08 ± 27.95	-2.20 ± 28.94	
	P-value[b]	0.2842	0.7728	0.3166
$PPG_{1.5h}$ (mg/dL)	Baseline	197.83 ± 26.53	186.53 ± 23.83	0.2552
	6 weeks	199.67 ± 32.75	181.20 ± 31.26	
	Variation from baseline	1.83 ± 26.60	-5.33 ± 42.59	
	P-value[b]	0.8157	0.6352	0.6159
	12 weeks	200.83 ± 36.75	187.27 ± 32.14	
	Variation from baseline	3.00 ± 28.90	0.73 ± 21.12	
	P-value[b]	0.7260	0.8949	0.8157
$PPG_{2.0h}$ (mg/dL)	Baseline	171.58 ± 14.62	161.13 ± 15.38	0.0851
	6 weeks	173.17 ± 26.86	162.73 ± 29.13	
	Variation from baseline	1.58 ± 25.47	1.60 ± 33.60	
	P-value[b]	0.8334	0.8563	0.9989
	12 weeks	175.33 ± 37.44	173.13 ± 21.08	
	Variation from baseline	3.75 ± 34.03	12.00 ± 23.61	
	P-value[b]	0.7099	0.0691	0.4643
Glucose $iAUC_{0-2h}$ (h*mg/dL)	Baseline	155.69 ± 41.43	155.62 ± 29.26	0.9959
	6 weeks	160.73 ± 41.63	145.77 ± 39.26	

Variation from baseline	5.04 ± 29.13	-9.85 ± 48.43	
P-value[b]	0.5610	0.4440	0.3581
12 weeks	163.90 ± 31.26	159.02 ± 28.43	
Variation from baseline	8.21 ± 22.32	3.40 ± 23.29	
P-value[b]	0.2289	0.5808	0.5920

FPG, fasting plasma glucose; PPG, postprandial plasma glucose; iAUC, incremental area under the curve; OGTT, oral glucose tolerance test. Values are expressed as the mean ± SD. *$P < 0.05$. [a] Compared between groups; *P*-value using the independent *t*-test. [b] Compared within groups; *P*-value using the paired *t*-test. †*P*-value was adjusted for baseline

Table S5-1. Fasting insulin, C-peptide, and insulin levels in the 75 g OGTT in 30–40-year-olds.

		Control group (n = 14)	Test group (n = 10)	*P*-value[a]
Fasting insulin (μU/mL)	Baseline	12.07 ± 6.04	15.25 ± 8.17	0.2841
	6 weeks	10.61 ± 5.18	11.64 ± 5.68	
	Variation from baseline	-1.46 ± 4.78	-3.61 ± 6.06	
	P-value[b]	0.2743	0.0922	0.3407
	12 weeks	12.36 ± 7.58	11.54 ± 5.56	
	Variation from baseline	0.29 ± 4.86	-3.71 ± 6.88	
	P-value[b]	0.8250	0.1223	0.1079
$Insulin_{0.5h}$ (μU/mL)	Baseline	82.35 ± 56.05	62.67 ± 34.99	0.3382
	6 weeks	94.16 ± 63.87	73.80 ± 49.91	
	Variation from baseline	11.81 ± 63.67	11.13 ± 23.98	
	P-value[b]	0.4997	0.1762	0.9711
	12 weeks	79.99 ± 49.04	51.76 ± 21.18	
	Variation from baseline	-2.36 ± 34.60	-10.91 ± 37.76	
	P-value[b]	0.8022	0.3847	0.5715
$Insulin_{1.0h}$ (μU/mL)	Baseline	96.99 ± 73.25	83.71 ± 42.44	0.6129
	6 weeks	90.44 ± 49.19	93.97 ± 46.39	
	Variation from baseline	-6.55 ± 68.43	10.26 ± 28.00	
	P-value[b]	0.7260	0.2764	0.4187
	12 weeks	109.61 ± 64.74	76.84 ± 22.70	
	Variation from baseline	12.62 ± 43.53	-6.87 ± 33.80	
	P-value[b]	0.2977	0.5364	0.2500
$Insulin_{1.5h}$ (μU/mL)	Baseline	107.97 ± 78.69	98.91 ± 45.21	0.7472
	6 weeks	106.86 ± 76.29	113.49 ± 53.22	
	Variation from baseline	-1.11 ± 66.90	14.58 ± 46.38	
	P-value[b]	0.9513	0.3461	0.5298
	12 weeks	107.69 ± 61.19	102.88 ± 66.34	
	Variation from baseline	-0.28 ± 32.65	3.97 ± 78.55	
	P-value[b]	0.9750	0.8766	0.8747
$Insulin_{2.0h}$ (μU/mL)	Baseline	116.28 ± 78.44	97.92 ± 36.22	0.4514
	6 weeks	103.49 ± 77.61	97.88 ± 33.19	
	Variation from baseline	-12.79 ± 71.02	-0.04 ± 42.27	
	P-value[b]	0.5124	0.9977	0.6184
	12 weeks	108.53 ± 72.94	83.29 ± 43.91	
	Variation from baseline	-7.75 ± 43.18	-14.63 ± 44.82	
	P-value[b]	0.5136	0.3289	0.7084
C-peptide (ng/mL)	Baseline	2.45 ± 0.78	2.53 ± 0.78	0.8076
	6 weeks	2.80 ± 1.01	2.33 ± 0.57	
	Variation from baseline	0.35 ± 0.76	-0.21 ± 0.43	
	P-value[b]	0.1126	0.1691	0.0519
	12 weeks	2.99 ± 0.97	2.47 ± 0.55	
	Variation from baseline	0.54 ± 0.87	-0.06 ± 0.52	
	P-value[b]	0.0378	0.7127	0.0655

OGTT, oral glucose tolerance test. Data are expressed as the mean ± SD. [a]Comparison between groups; *P*-value determined using the independent *t*-test. [b]Comparison within groups; *P*-value determined using the paired *t*-test.

Table S5-2. Fasting insulin, C-peptide, and insulin levels in the 75 g OGTT in 50–59-year-olds.

		Control group (n = 12)	Test group (n = 12)	*P*-value[a]
Fasting insulin (μU/mL)	Baseline	7.76 ± 3.78	9.08 ± 5.83	0.5184
	6 weeks	8.23 ± 4.81	11.41 ± 8.86	
	Variation from baseline	0.47 ± 2.57	2.33 ± 9.53	0.5242
	P-value[b]	0.5419	0.4145	
	12 weeks	8.43 ± 3.60	7.49 ± 3.34	
	Variation from baseline	0.67 ± 2.93	-1.58 ± 3.66	
	P-value[b]	0.4479	0.1625	0.1110
$Insulin_{0.5h}$ (μU/mL)	Baseline	56.52 ± 51.00	51.00 ± 27.90	0.7454
	6 weeks	56.06 ± 32.04	54.58 ± 39.57	
	Variation from baseline	-0.46 ± 42.14	3.58 ± 26.10	
	P-value[b]	0.9706	0.6437	0.7802
	12 weeks	62.28 ± 43.21	41.78 ± 37.89	
	Variation from baseline	5.77 ± 15.68	-9.23 ± 17.19	
	P-value[b]	0.2289	0.0900	**0.0361***
$Insulin_{1.0h}$ (μU/mL)	Baseline	63.13 ± 39.33	70.48 ± 33.84	0.6285
	6 weeks	63.40 ± 49.24	77.35 ± 41.07	
	Variation from baseline	0.27 ± 29.51	6.87 ± 39.05	
	P-value[b]	0.9756	0.5548	0.6450
	12 weeks	66.68 ± 28.61	61.51 ± 30.22	
	Variation from baseline	3.54 ± 32.11	-8.98 ± 26.06	
	P-value[b]	0.7097	0.2580	0.3058
$Insulin_{1.5h}$ (μU/mL)	Baseline	60.83 ± 22.16	73.31 ± 23.65	0.1958
	6 weeks	77.29 ± 37.00	65.68 ± 37.24	
	Variation from baseline	16.47 ± 25.91	-7.63 ± 30.02	
	P-value[b]	0.0499	0.3973	**0.0469***
	12 weeks	70.17 ± 30.99	72.82 ± 35.47	
	Variation from baseline	9.34 ± 25.78	-0.49 ± 36.22	
	P-value[b]	0.2355	0.9633	0.4517
$Insulin_{2.0h}$ (μU/mL)	Baseline	72.88 ± 38.83	75.90 ± 30.39	0.8337
	6 weeks	77.25 ± 48.38	81.82 ± 51.98	
	Variation from baseline	4.37 ± 43.50	5.92 ± 52.34	
	P-value[b]	0.7341	0.7028	0.9382
	12 weeks	77.30 ± 38.45	86.03 ± 48.19	
	Variation from baseline	4.42 ± 31.77	10.13 ± 45.82	
	P-value[b]	0.6389	0.4597	0.7262
C-peptide (ng/mL)	Baseline	2.09 ± 0.83	2.21 ± 0.64	0.6900
	6 weeks	2.20 ± 0.98	2.27 ± 0.51	
	Variation from baseline	0.11 ± 0.42	0.06 ± 0.48	
	P-value[b]	0.3950	0.6651	0.8031
	12 weeks	2.33 ± 0.72	2.18 ± 0.73	
	Variation from baseline	0.24 ± 0.53	-0.03 ± 0.46	
	P-value[b]	0.1444	0.8052	0.1913

OGTT, oral glucose tolerance test. Data are expressed as the mean ± SD. *$P < 0.05$. [a]Comparison between groups; [b]Comparison within groups; *P*-value determined using the paired *t*-test.

Table S5-3. Fasting insulin, C-peptide, and insulin levels in the 75 g OGTT in 60–69-year-olds.

		Control group (n = 12)	Test group (n = 15)	*P*-value[a]
Fasting insulin (μU/mL)	Baseline	8.59 ± 4.10	9.19 ± 5.59	0.7608
	6 weeks	6.22 ± 2.52	7.23 ± 3.45	
	Variation from baseline	-2.37 ± 2.84	-1.96 ± 3.18	
	P-value[b]	0.0144	0.0315	0.7268
	12 weeks	6.89 ± 4.62	6.83 ± 3.96	
	Variation from baseline	-1.70 ± 3.45	-2.35 ± 3.08	
	P-value[b]	0.1155	0.0103	0.6078
$Insulin_{0.5h}$ (μU/mL)	Baseline	52.03 ± 32.68	52.19 ± 24.02	0.9878
	6 weeks	45.91 ± 29.71	55.13 ± 34.22	
	Variation from baseline	-6.12 ± 19.75	2.93 ± 21.81	0.2748
	P-value[b]	0.3064	0.6105	
	12 weeks	43.48 ± 31.79	50.76 ± 24.08	
	Variation from baseline	-8.54 ± 32.57	-1.43 ± 10.03	
	P-value[b]	0.3832	0.5888	0.4794
$Insulin_{1.0h}$ (μU/mL)	Baseline	77.93 ± 36.06	83.11 ± 38.05	0.7221
	6 weeks	64.26 ± 44.65	68.25 ± 43.22	
	Variation from baseline	-13.67 ± 33.12	-14.85 ± 18.19	
	P-value[b]	0.1806	0.0069	0.9127
	12 weeks	77.10 ± 47.15	70.78 ± 42.13	
	Variation from baseline	-0.83 ± 58.13	-12.33 ± 35.33	
	P-value[b]	0.9617	0.1981	0.5311
$Insulin_{1.5h}$ (μU/mL)	Baseline	102.86 ± 51.17	87.71 ± 48.53	0.4389
	6 weeks	76.84 ± 33.34	74.26 ± 36.83	
	Variation from baseline	-26.02 ± 32.45	-13.45 ± 48.23	
	P-value[b]	0.0180	0.2982	0.4474
	12 weeks	79.30 ± 42.91	78.81 ± 51.39	
	Variation from baseline	-23.56 ± 35.13	-8.90 ± 30.46	
	P-value[b]	0.0403	0.2768	0.2566
$Insulin_{2.0h}$ (μU/mL)	Baseline	86.79 ± 38.81	87.39 ± 56.53	0.9752
	6 weeks	84.99 ± 55.23	72.29 ± 40.07	
	Variation from baseline	-1.80 ± 50.83	-15.10 ± 55.23	0.5256
	P-value[b]	0.9046	0.3076	
	12 weeks	82.48 ± 46.38	90.99 ± 72.99	
	Variation from baseline	-4.31 ± 29.34	3.59 ± 35.26	
	P-value[b]	0.6210	0.6990	0.5394
C-peptide (ng/mL)	Baseline	2.31 ± 0.67	2.12 ± 0.81	0.5255
	6 weeks	2.12 ± 0.60	2.07 ± 0.66	
	Variation from baseline	-0.19 ± 0.19	-0.05 ± 0.62	
	P-value[b]	0.0059	0.7458	0.4356
	12 weeks	2.12 ± 0.72	1.90 ± 0.64	
	Variation from baseline	-0.19 ± 0.40	-0.22 ± 0.56	
	P-value[b]	0.1352	0.1467	0.8643

OGTT, oral glucose tolerance test. Data are expressed as the mean ± SD. [a]Comparison between groups; *P*-value determined using the independent *t*-test. [b]Comparison within groups; *P*-value determined using the paired *t*-test.

Table S6-1. Insulin sensitivity surrogate markers in 30–40-year-olds.

		Control group (n = 14)	Test group (n = 10)	*P*-value[a]
HOMA-IR	Baseline	2.94 ± 1.50	3.93 ± 2.26	0.2091
	6 weeks	4.06 ± 3.28	2.96 ± 1.19	
	Variation from baseline	1.12 ± 2.48	-0.97 ± 1.76	
	P-value[b]	0.1149	0.1157	**0.0328***
	12 weeks	3.96 ± 2.15	3.49 ± 1.92	
	Variation from baseline	1.02 ± 1.93	-0.44 ± 1.90	
	P-value[b]	0.0708	0.4795	0.0798
HOMA-β (%)	Baseline	126.27 ± 65.68	142.89 ± 64.03	0.5432
	6 weeks	159.32 ± 74.07	113.22 ± 42.95	
	Variation from baseline	33.05 ± 78.67	-29.67 ± 61.66	
	P-value[b]	0.1400	0.1624	**0.0476***
	12 weeks	156.84 ± 80.04	111.61 ± 30.43	
	Variation from baseline	30.58 ± 70.07	-31.28 ± 47.60	
	P-value[b]	0.1265	0.0675	**0.0245***
QUICKI	Baseline	0.33 ± 0.03	0.32 ± 0.03	0.3529
	6 weeks	0.32 ± 0.03	0.33 ± 0.02	
	Variation from baseline	-0.01 ± 0.02	0.01 ± 0.02	
	P-value[b]	0.0475	0.1081	**0.0115***
	12 weeks	0.32 ± 0.03	0.33 ± 0.03	
	Variation from baseline	-0.01 ± 0.02	0.00 ± 0.02	
	P-value[b]	0.0480	0.7128	0.1200
$ISI_{stumvoll}$	Baseline	0.106 ± 0.018	0.106 ± 0.015	0.9674
	6 weeks	0.106 ± 0.019	0.108 ± 0.014	
	Variation from baseline	0.000 ± 0.005	0.002 ± 0.004	
	P-value[b]	0.8973	0.0721	0.1912
	12 weeks	0.105 ± 0.019	0.109 ± 0.015	
	Variation from baseline	-0.001 ± 0.004	0.003 ± 0.005	
	P-value[b]	0.5088	0.0828	0.0532
$ISI_{0,120}$	Baseline	35.08 ± 10.37	32.96 ± 6.60	0.5761
	6 weeks	37.20 ± 9.92	33.04 ± 5.62	
	Variation from baseline	2.12 ± 9.90	0.07 ± 4.79	
	P-value[b]	0.4367	0.9627	0.5092
	12 weeks	36.74 ± 12.60	35.97 ± 8.71	
	Variation from baseline	1.66 ± 6.25	3.01 ± 5.13	
	P-value[b]	0.3387	0.0963	0.5804
HbA1c (%)	Baseline	5.58 ± 0.21	5.70 ± 0.31	0.2613
	12 weeks	5.54 ± 0.18	5.60 ± 0.30	
	Variation from baseline	-0.04 ± 0.16	-0.10 ± 0.14	
	p-value[‡]	0.3212	0.0522	0.3672

HOMA-IR, homeostatic model assessment for insulin resistance; HOMA-β, homeostatic model assessment of β-cell function; QUICKI, quantitative insulin sensitivity check index; ISI, insulin sensitivity index. Values are expressed as the mean ± SD *P < 0.05. [a]Comparison between groups: the *P*-value was determined using the independent *t*-test. [b]Comparison within groups: P-value was determined using the paired *t*-test.

Table S6-2. Insulin sensitivity surrogate markers in 50–59-year-olds.

		Control group (n = 12)	Test group (n = 12)	*P*-value[a]
HOMA-IR	Baseline	2.00 ± 1.07	2.26 ± 1.51	0.6217
	6 weeks	2.33 ± 1.70	2.40 ± 1.07	
	Variation from baseline	0.33 ± 1.07	0.14 ± 1.24	
	P-value[b]	0.3028	0.7028	0.6871
	12 weeks	2.84 ± 1.57	2.30 ± 1.17	
	Variation from baseline	0.84 ± 1.34	0.03 ± 1.21	
	P-value[b]	0.0526	0.9236	0.1360
HOMA-β (%)	Baseline	75.41 ± 38.99	87.53 ± 52.23	0.5263
	6 weeks	77.56 ± 34.74	88.14 ± 37.46	
	Variation from baseline	2.15 ± 30.37	0.61 ± 54.39	
	P-value[b]	0.8111	0.9697	0.9327
	12 weeks	86.04 ± 24.44	84.60 ± 40.11	
	Variation from baseline	10.63 ± 36.50	-2.92 ± 47.60	
	P-value[b]	0.3347	0.8355	0.4422
QUICKI	Baseline	0.35 ± 0.03	0.35 ± 0.03	0.6000
	6 weeks	0.35 ± 0.03	0.34 ± 0.02	
	Variation from baseline	-0.01 ± 0.02	-0.01 ± 0.02	
	P-value[b]	0.4161	0.2179	0.7927
	12 weeks	0.33 ± 0.02	0.34 ± 0.03	
	Variation from baseline	-0.02 ± 0.03	0.00 ± 0.02	
	P-value[b]	0.0468	0.6380	0.1371
$ISI_{stumvoll}$	Baseline	0.112 ± 0.011	0.113 ± 0.015	0.9505
	6 weeks	0.113 ± 0.011	0.111 ± 0.015	
	Variation from baseline	0.000 ± 0.002	-0.002 ± 0.005	
	P-value[b]	0.8321	0.2113	0.2070
	12 weeks	0.112 ± 0.011	0.114 ± 0.015	
	Variation from baseline	0.000 ± 0.003	0.001 ± 0.003	
	P-value[b]	0.9167	0.2631	0.3543
$ISI_{0,120}$	Baseline	36.73 ± 5.87	37.52 ± 5.87	0.7459
	6 weeks	34.80 ± 7.38	53.87 ± 64.41	
	Variation from baseline	-1.93 ± 5.46	16.35 ± 64.14	
	P-value[b]	0.2452	0.3961	0.3460
	12 weeks	35.13 ± 8.73	53.82 ± 63.75	
	Variation from baseline	-1.60 ± 7.91	16.30 ± 63.49	
	P-value[b]	0.4976	0.3927	0.3525
HbA1c (%)	Baseline	5.74 ± 0.45	5.83 ± 0.28	0.5543
	12 weeks	5.71 ± 0.41	5.86 ± 0.29	
	Variation from baseline	-0.03 ± 0.16	0.02 ± 0.11	0.2943
	p-value[‡]	0.4739	0.4293	0.2943

HOMA-IR, homeostatic model assessment for insulin resistance; HOMA-β, homeostatic model assessment of β-cell function; QUICKI, quantitative insulin sensitivity check index; ISI, insulin sensitivity index. Values are expressed as the mean ± SD [a]Comparison between groups: the *P*-value was determined using the independent *t*-test. [b]Comparison within groups: *P*-value was determined using the paired *t*-test.

Table S6-3. Insulin sensitivity surrogate markers in 60–69-year-olds.

		Control group (n = 12)	Test group (n = 15)	*P*-value[a]
HOMA-IR	Baseline	2.13 ± 0.95	2.23 ± 1.41	0.8499
	6 weeks	1.84 ± 0.59	1.84 ± 0.86	
	Variation from baseline	-0.30 ± 0.63	-0.38 ± 1.16	
	P-value[b]	0.1332	0.2246	0.8080
	12 weeks	1.99 ± 0.80	1.79 ± 0.95	
	Variation from baseline	-0.15 ± 0.54	-0.44 ± 1.20	
	P-value[b]	0.3571	0.1801	0.4145
HOMA-β (%)	Baseline	85.82 ± 50.74	96.57 ± 52.21	0.5951
	6 weeks	75.13 ± 28.13	84.14 ± 27.88	
	Variation from baseline	-10.69 ± 36.13	-12.43 ± 35.07	
	P-value[b]	0.3276	0.1914	0.9001
	12 weeks	81.43 ± 37.20	81.25 ± 36.81	
	Variation from baseline	-4.39 ± 26.88	-15.32 ± 39.60	
	P-value[b]	0.5833	0.1562	0.4219
QUICKI	Baseline	0.35 ± 0.03	0.35 ± 0.04	0.6533
	6 weeks	0.35 ± 0.02	0.36 ± 0.03	
	Variation from baseline	0.00 ± 0.02	0.00 ± 0.03	
	P-value[b]	0.7090	0.8657	0.9120
	12 weeks	0.35 ± 0.02	0.36 ± 0.04	
	Variation from baseline	0.00 ± 0.02	0.01 ± 0.03	
	P-value[b]	0.6671	0.4016	0.6573
$ISI_{stumvoll}$	Baseline	0.106 ± 0.013	0.108 ± 0.011	0.6306
	6 weeks	0.107 ± 0.013	0.110 ± 0.010	
	Variation from baseline	0.001 ± 0.002	0.002 ± 0.002	
	P-value[b]	0.0865	0.0019	0.3633
	12 weeks	0.107 ± 0.013	0.111 ± 0.010	
	Variation from baseline	0.001 ± 0.003	0.002 ± 0.002	
	P-value[b]	0.2980	<.0001	0.1833
$ISI_{0,120}$	Baseline	33.01 ± 7.20	35.27 ± 8.69	0.4772
	6 weeks	34.59 ± 8.03	38.23 ± 9.61	
	Variation from baseline	1.58 ± 5.95	2.96 ± 7.67	
	P-value[b]	0.3776	0.1574	0.6139
	12 weeks	32.99 ± 7.27	35.15 ± 9.29	
	Variation from baseline	-0.02 ± 4.44	-0.12 ± 4.63	
	P-value[b]	0.9878	0.9228	0.9561
HbA1c (%)	Baseline	5.83 ± 0.24	5.78 ± 0.25	0.5778
	12 weeks	5.83 ± 0.15	5.71 ± 0.28	
	Variation from baseline	-0.01 ± 0.17	-0.07 ± 0.14	
	p-value[‡]	0.8705	0.0859	0.3412

HOMA-IR, homeostatic model assessment for insulin resistance; HOMA-β, homeostatic model assessment of β-cell function; QUICKI, quantitative insulin sensitivity check index; ISI, insulin sensitivity index. Values are expressed as the mean ± SD [a]Comparison between groups: the *P*-value was determined using the independent *t*-test. [b]Comparison within groups: *P*-value was determined using the paired *t*-test.
